# Need for Supplemental Oxygen at Discharge in Infants with Bronchopulmonary Dysplasia Is Not Associated with Worse Neurodevelopmental Outcomes at 3 Years Corrected Age

**DOI:** 10.1371/journal.pone.0090843

**Published:** 2014-03-19

**Authors:** Abhay Lodha, Reg Sauvé, Vineet Bhandari, Selphee Tang, Heather Christianson, Anita Bhandari, Harish Amin, Nalini Singhal

**Affiliations:** 1 Department of Pediatrics, Foothills Medical Centre, Peter Lougheed Centre, Alberta Children’s Hospital, Calgary, Canada; 2 Community Health Sciences, University of Calgary, Calgary, Canada; 3 Alberta Health Services, Calgary, Canada; 4 Alberta Children’s Hospital Institute of Child & Maternal Health, Calgary, Canada; 5 Department of Pediatrics, Yale University School of Medicine, New Haven, Connecticut, United States of America; 6 Department of Pediatric Pulmonology, Connecticut Children’s Medical Center, Hartford, Connecticut, United States of America; University of British Columbia, Canada

## Abstract

**Objectives:**

To determine if chronic oxygen dependency (discharge home on supplemental oxygen) in children with bronchopulmonary dysplasia (BPD; defined as requirement for supplemental O_2_ at 36 weeks postmenstrual age) predicts neurodevelopmental disability rates and growth outcomes at 36 months corrected age (CA).

**Study Design:**

Longitudinal cohort study.

**Setting:**

Southern Alberta regional center located at high altitude.

**Participants:**

Preterm infants weighing ≤1250 grams with no BPD, BPD, and BPD with chronic oxygen dependency.

**Main outcome measures:**

Neurodevelopmental and growth outcomes.

**Results:**

Of 1563 preterm infants admitted from 1995–2007, 1212 survived. Complete follow-up data were available for 1030 (85%) children. Children in BPD and BPD with chronic oxygen dependency groups had significantly lower birth weights, gestational ages, prolonged mechanical ventilation and oxygen supplementation and received more postnatal steroids, compared to those without BPD. Children with BPD and BPD with chronic oxygen dependency were more likely to be below the 5^th^ centile in weight and height compared to those without BPD but there was little difference between the BPD and BPD with chronic oxygen dependency groups. After controlling for confounding variables, children who had BPD and BPD with chronic oxygen dependency had higher odds of neurodevelopmental disability compared to those without BPD [OR (odds ratio) 1.9 (95%CI 1.1 to 3.5) and OR 1.8 (1.1 to 2.9), respectively], with no significant difference between BPD and BPD with chronic oxygen dependency [OR 0.9 (95% CI 0.6 to 1.5)].

**Conclusions:**

BPD and BPD with chronic oxygen dependency in children predicts abnormal neurodevelopmental outcomes at 36 months CA. However, the neurodevelopmental disability rates were not significantly higher in BPD with chronic oxygen dependency children compared to children with BPD only. Compared to those without BPD, growth is impaired in children with BPD and BPD with chronic oxygen dependency, but no difference between the latter two groups.

## Introduction

Bronchopulmonary dysplasia (BPD) is a common and serious problem in very preterm infants. It is characterized by early lung injury and can progress to severe BPD [1]. The incidence of BPD in preterm infants has varied from 4.6% to 72% depending on birth weight and gestational age category, definitions used [2], neonatal assisted ventilation strategies [3] and site of care [2], [3]. Shannon et al. [4] defined BPD based on oxygen dependency at 36 weeks postmenstrual age (PMA) as opposed to Northway’s definition [5] of BPD based on oxygen requirement at 28 days of life. This change in definition based on longer duration of oxygen requirement has had an impact on predicting preterm infants’ long term neurodevelopmental outcomes. Frequent episodes of hypoxemia in BPD infants may affect growth, cardiac functions and long term neurodevelopmental outcomes [6]. Therefore, the purpose of home oxygen therapy is to prevent the effects of hypoxemia and to prevent pulmonary and bronchial vasoconstriction leading to alteration in the airway causing obstruction and impairment in growth of pulmonary and ocular vasculature and their effects on long term neurodevelopmental outcomes [3], [7]–[9].

In a study by Majnemer et al., it was found that preterm infants with BPD who required home oxygen therapy were at a greater risk of poor neurodevelopmental outcomes at school age [10]. In a recent study, Trittmann et al. revealed that at 18 months corrected age, the need for supplemental oxygen at discharge was not associated with an increased risk of neurodevelopmental disability [11]. However, there are no reports of large longitudinal single center studies that examine the long term impact of chronic oxygen dependency in preterm infants with BPD, differentiating between infants with and without chronic oxygen dependency, on the risk for adverse neurodevelopmental outcomes at 36 months corrected age (CA).

We hypothesized that for preterm infants, the incidence of poor growth, neurodevelopmental disability, and abnormal language outcomes increases as the severity of BPD increases from no BPD, to BPD, and BPD with chronic oxygen dependency.

The primary objective of this study was to determine the relationship between BPD severity in preterm infants based on chronic oxygen dependency and neurodevelopmental disability rates. Secondary objectives examined the relationship between BPD severity with growth and language outcomes at 36 months CA.

## Methods

Premature infants with birth weight ≤1250 grams born between January 1995 and December 2007 and admitted to the single largest tertiary Neonatal Intensive Care Unit (NICU) in Southern Alberta were included in the study. These infants were followed longitudinally from birth at a regional Perinatal Follow-up Program that serves the geographic catchment area of Southern Alberta, Canada. Infants with major congenital malformations or chromosomal disorders were excluded from this study. This study was approved by the institutional ethics review board of the University of Calgary, and signed consent was obtained from the parents of all study participants.

Standardized demographic, perinatal and neonatal data were collected from patients’ charts by a by research coordinator and entered into a computerized database when the premature infants were discharged from the NICU. Occupations of fathers were ranked according to the Blishen socioeconomic index for occupations in Canada [12].

The specific guidelines for discharging an infant home from NICU are presented in Table 1. According to Alberta Health Provincial guidelines (page no. 34), a dated hard copy of pulse oximetry results 48 hours before discharge showing room air SpO2≤89% was required to discharge the baby home on oxygen. http://www.health.alberta.ca/documents/AADL-Manual-R-Respiratory.pdf.

**Table 1:** Discharge criteria of infant from NICU.

Criteria
a. Corrected gestational age >34 weeks
b. Able to maintain body temperature (>36.4°C) in an open crib
c. Competent suckle feeding (breast or bottle) without cardiorespiratory compromise and gaining weight
d. Physiologically mature
e. Stable cardiorespiratory function
f. No apnea of prematurity
g. No active medical problems
h. Receipt of appropriate immunizations
i. Appropriate metabolic screening and car seat testing performed
j. In addition to the above criteria, family readiness

At discharge the infants were categorized as having no BPD, BPD (O_2_ dependency at 36 weeks PMA but not at the time of discharge home), and BPD with chronic oxygen dependency (O_2_ supplementation at 36 weeks PMA and discharged home on O_2_). We have had the same research coordinator in our Southern Alberta Perinatal follow-up program for the last 30 years and she has been consistently collecting the data from patients’ charts and entering them into the database. Outcome definitions did not change during the study period in our perinatal follow-up clinic.

Perinatal and neonatal data were defined according to the Canadian Neonatal Network manual. (http://www.canadianneonatalnetwork.org/Portal/LinkClick.aspx?fileticket=I3jnvN9fGfE%3D&tabid=69) Gestational age (GA) was defined as the best estimate based on obstetric history, obstetric examination and first prenatal ultrasound examination [13]. Bronchopulmonary dysplasia was defined as supplemental oxygen utilization at 36 weeks PMA [4]. Diagnosis of patent ductus arteriosus (PDA) was made clinically, with or without echocardiography [13]. Intraventricular hemorrhage (IVH) was diagnosed and classified based on the Canadian Pediatric Society’s Cranial Ultrasound Statement [14] . Severe neurological injury defined as the presence of grade 3 or 4 intraventricular hemorrhage (IVH) or parenchymal echolucency [13]. Retinopathy of prematurity (ROP) grade 3 or 4 was defined according to the International Classification for ROP [13]. Necrotizing enterocolitis (NEC) was defined according to Bell’s criteria (stage≥2) [13]. Small for gestational age (SGA) was defined as birth weight <10^th^ percentile for the given GA [13]. Duration of mechanical ventilation was defined as the total number of days during which the infant was on mechanical ventilation during any part of the day. Total duration of oxygen use was defined as the total number of days during which the infant received supplemental oxygen. Length of stay was defined as the total number of days that an infant stayed in the NICU. These definitions remained constant throughout the study period.

All surviving preterm infants were routinely followed prospectively and underwent comprehensive developmental assessment by a multidisciplinary team (consisting of a neonatologist/developmental pediatrician, psychologist, occupational therapist, physiotherapist, dietician, speech therapist, social worker, nurse, ophthalmologist, audiologist) at 4, 8, and 18 and at 36 months CA, with referrals to treatment made as required. The cognitive assessments were performed by a trained psychologist using the Wechsler Preschool and Primary Scale of Intelligence, Third Edition (WPPSI-III) or the Stanford-Binet Intelligence Test, Fourth Edition (SB-IV). Formal speech and language assessments were completed by a speech and language pathologist using a battery of standardized test measures including the Preschool Language Scale (PLS 3 or 4) and the Clinical Evaluation of Language Fundamentals (CELF-P2). An audiologist examined each child using visual reinforcement audiometry, otoscopic evaluation and tympanometry at 4 months of CA. Members of the multidisciplinary team were not blinded to the details of the infant’s neonatal hospital course.

For the primary outcome, neurodevelopmental disability at 36 months CA was classified using three categories: i) no disability, ii) mild disability, and iii) severe disability.

### Classification of Disability

Disability was classified into two groups based on severity of disability: mild or severe. Mild disability was considered present if a child had one or more of the following conditions: mild, ambulant cerebral palsy, borderline cognitive scores of 1–2 standard deviations (SD) below the mean on standardized tests, visual or hearing disabilities, but were not blind or deaf. Severe disability was defined as one or more of: moderate-severe cerebral palsy, cognitive score >2 SD below the mean on standardized tests, blindness or deafness.

Detailed definitions of disability outcomes are described below.

#### Cerebral palsy

(CP) refers to a non-progressive disability of movement and posture and was diagnosed on the basis of abnormal muscle tone and reflexes on the physical and neurological examination. Severity of motor dysfunction in cerebral palsy was classified into two categories: mild or moderate-severe. Mild CP was defined as having abnormal tone and reflexes with no limiting effects on daily activities and functions. Moderate-severe CP was defined as motor dysfunction requiring appliances or assistance with performance of daily activities and functions [15].

#### Cognitive delay

Delayed cognitive function was diagnosed if there was a cognitive score >2 SD below the mean on age appropriate standardized testing. Borderline cognitive function was diagnosed if there was a cognitive score 1–2 SD below the mean on age appropriate standardized testing.

#### Blindness

was considered present if the infant had bilateral blindness with corrected visual acuity of <20/200 in the better eye. Mild vision disability refers to those infants who had corrected visual acuity <20/60 but >20/200 in the better eye, significant refractive errors such as severe myopia or significant hypermetropia, or unilateral blindness.

#### Deafness

was defined as a bilateral sensorineural loss requiring amplification or cochlear implants. Mild hearing disability was defined as neurosensory hearing loss not requiring amplification or implants, or unilateral hearing loss requiring amplification.

### Secondary Outcomes

#### Poor growth

Poor growth was considered present if weight, height, or head circumference was <5^th^ centile at 36 months CA based on the Centers for Disease Control and Prevention (CDC 2000) growth curves [16].

#### Abnormal language

Overall abnormal communication was defined as receptive, expressive, or overall language scores >1 SD below the mean on standardized language tests, or if articulation was unintelligible. Abnormal receptive or expressive language was based on a score of >1 SD below the mean on the receptive or expressive component of standardized language tests respectively.

### Statistical Analysis

The sample size estimation was based on the primary objective of this study: to examine the relationship between no BPD, BPD, and BPD with chronic oxygen dependency and neurodevelopmental disability. We used the method derived by Whitehead [17] for the estimation of sample size for ordinal data, where the cumulative odds ratio is the effect size. The cumulative odds ratio is calculated using the cumulative probability. In this case, the dependent variable consisted of three categories: no, mild, and severe disability. Thus, the two cumulative probabilities are the probability of any disability (mild or severe) and the probability of severe disability. The two cumulative odds are the probability of any disability divided by the probability of no disability and the probability of severe disability divided by the probability of mild or no disability. In order to detect an ordinal odds ratio of at least 2.0 with alpha = 0.05 and 80% power, we estimated a sample size of at least 120 children in each of the three categories. Our total sample size was 1030 eligible infants, which allowed us to adjust for covariates.

The infant and maternal characteristics of the cohort were compared across the three groups using Pearson Chi-square or Fisher’s exact test for categorical variables, and, since they were not normally-distributed with homogeneous variances, the Kruskal-Wallis test for continuous variables. To assess the three pairwise differences between groups (no BPD vs. BPD, no BPD vs. BPD with chronic oxygen dependency, and BPD vs. BPD with chronic oxygen dependency), confidence intervals for differences in proportion or Hodges-Lehmann median differences were calculated. To adjust for multiple comparisons, the Bonferroni correction was applied, and confidence intervals reported at the (1–0.05/3) level.

Given that our primary outcome was ordinal, a proportional odds model was used to determine the effect of the three levels of BPD status (independent variable) on neurodevelopmental disability (dependent variable). This approach considers the scale over the levels of disability and models the ‘odds of greater disability’ (the odds of mild or severe disability versus no disability, and the odds of severe disability versus mild or no disability). Candidate covariates were identified based on the association with BPD status from the univariate analyses. Covariates considered were: maternal race, caesarean section, maternal antihypertensive medications use, GA, birth weight (BW), sex, small for gestational age (SGA), duration of neonatal hospital stay, days on ventilation, days on oxygen, number of blood transfusions, postnatal steroids, diuretics, PDA, respiratory distress syndrome (RDS), sepsis, NEC, IVH, and ROP. In order to determine the most parsimonious model, we started with a model with all candidate variables included, and any non-statistically significant variables at the alpha = 0.05 level, aside from BPD status, were considered for elimination. The likelihood ratio test and Akaike’s Information Criterion were used to assess whether a variable could be removed from the model without significantly affecting model fit. Once the final model was obtained, we included GA and BW separately, as these are clinically important covariates. The Score test was used to test the proportional odds assumption of the final model.

Secondary outcomes of poor growth and abnormal language were explored using the Chi-square test to compare the three groups, along with the Bonferroni-corrected confidence intervals to assess pairwise differences between groups. Growth z-scores were compared across the three groups using ANOVA. All results were generated using SAS 9.3 (SAS Institute, Cary, NC, USA) and a significance level of 0.05 was used for all analyses.

## Results

**Figure 1:**
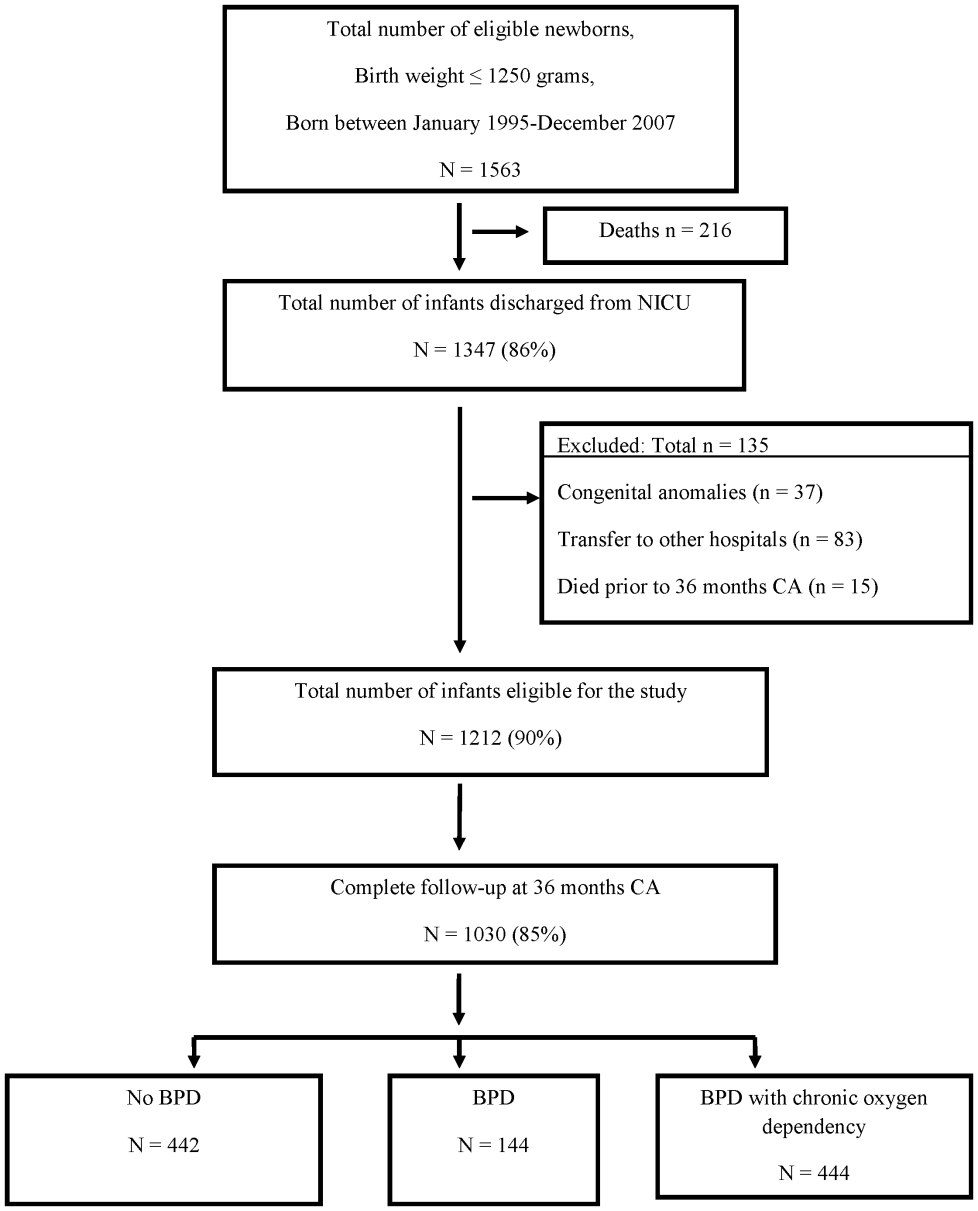
Flow diagram of the study cohort.

A total of 1563 infants weighing ≤1250 g were eligible at birth for study entry. A flow diagram of our study cohort is shown in **Figure 1**. After excluding infants who died prior to 36 months CA, congenital anomalies and unknown BPD status due to transfers to other hospitals, a total of 1212 children were eligible for study entry. In this cohort, almost 90% of children were seen at least once over the three year follow-up period but at 36 months CA the follow-up rate was reduced to 85% (1030 children). These children were divided into three groups: 442 (43%) with no BPD, 144 (14%) with BPD, and 444 (43%) with BPD with chronic oxygen dependency.

Demographic characteristics and socio-economic status of the study population are shown in **Table 2**. Children lost to follow-up at 36 months CA of age (n = 182) and those followed up (n = 1030) were not significantly different in BW (median 983 vs. 950 g, p = 0.101) or GA (median 28 vs. 27 weeks, p = 0.096). Children lost to follow-up were more likely to have a shorter hospital stay (median 71 vs. 75 days, p = 0.019) and duration of mechanical ventilation (median 6 vs. 13 days, p = 0.031). Follow-up rates were similar among the three groups of children.

**Table 2:** Characteristics of the Follow-up Cohort and Their Mothers.

Characteristic	No BPD	BPD	BPD withchronic oxygendependency	p-value†	Confidence Intervals forPairwise Differences*		
Infants	(n = 442)	(n = 144)	(n = 444)		No BPDvs. BPD	No BPD vs. BPDwith chronicoxygen dependency	BPD vs. BPDwith chronicoxygen dependency
Birth weight-grams, median (IQR)	1070 (930, 1180)	885 (750, 1060)	820 (690, 1000)	<0.001	(90 to 180)	(180 to 245)	(20 to 120)
Gestational age -wk, median (IQR)	29 (27, 30)	27 (26, 28)	26 (25, 28)	<0.001	(1 to 2)	(2 to 3)	(0 to 1)
Gestational age at discharge-wk, median (IQR)	37 (35, 38)	40 (38, 41)	39 (37, 41)	<0.001	(−4 to −2)	(−3 to −2)	(0 to 1)
Male sex, no. (%)	201 (45.5)	68 (47.2)	256 (57.7)	<0.001	(−0.13 to 0.10)	(−0.20 to −0.04)	(−0.22 to 0.01)
Singleton, no. (%)	311 (70.4)	90 (62.5)	317 (71.4)	0.121	(−0.03 to 0.19)	(−0.08 to 0.06)	(−0.20 to 0.02)
Inborn, no. (%)	396 (89.6)	124 (86.1)	391 (88.1)	0.496	(−0.04 to 0.11)	(−0.04 to 0.07)	(−0.10 to 0.06)
Small for gestational age (<10^th^ %ile), no. (%)	95 (21.5)	18 (12.5)	70 (15.9)	0.019	(0.01 to 0.17)	(−0.01 to 0.12)	(−0.11 to 0.04)
Surfactant, no. (%)	168 (38.2)	95 (66.0)	333 (75.2)	<0.001	(−0.39 to −0.17)	(−0.44 to −0.30)	(−0.20 to 0.01)
Positive pressure ventilation, (CMV+HFO) no. (%)	250 (56.6)	133 (92.4)	424 (95.5)	<0.001	(−0.44 to −0.28)	(−0.45 to −0.33)	(−0.09 to 0.03)
NCPAP, no. (%)	170 (38.5)	107 (74.3)	368 (82.9)	<0.001	(−0.46 to −0.26)	(−0.51 to −0.37)	(−0.18 to 0.01)
Hospital stay-days, median (IQR)	56 (43, 71)	93 (76, 103)	92 (74, 109)	<0.001	(−39 to −30)	(−39 to −32)	(−7 to 4)
Total ventilation-days, median (IQR)	1 (0, 6)	21 (8, 39)	36 (18, 52)	<0.001	(−20 to −12)	(−34 to −27)	(−17 to −6)
Total suppl. oxygen-days, median (IQR)	6 (2, 37)	78 (62, 93)	209 (153, 277)	<0.001	(−68 to −56)	(−200 to −177)	(−144 to −112)
Total blood transfusions-times, median (IQR)	0 (0, 1)	3 (1, 4)	4 (2, 6)	<0.001	(−3 to −2)	(−4 to −3)	(−2 to 0)
Postnatal steroids, no. (%)	21 (4.8)	29 (20.3)	144 (32.5)	<0.001	(−0.24 to −0.07)	(−0.34 to −0.22)	(−0.22 to −0.03)
Diuretics, no. (%)	139 (31.6)	114 (79.7)	384 (86.5)	<0.001	(−0.58 to −0.38)	(−0.61 to −0.48)	(−0.16 to 0.02)
Patent ductus arteriosus, no. (%)	117 (26.5)	89 (61.8)	307 (69.3)	<0.001	(−0.46 to −0.24)	(−0.50 to −0.36)	(−0.19 to 0.04)
Respiratory distress syndrome, no. (%)	224 (50.7)	123 (85.4)	386 (87.1)	<0.001	(−0.44 to −0.26)	(−0.43 to −0.30)	(−0.10 to 0.06)
Confirmed sepsis, no. (%)	42 (9.7)	39 (27.3)	110 (25.0)	<0.001	(−0.27 to −0.08)	(−0.21 to −0.09)	(−0.08 to 0.12)
Necrotizing enterocolitis, no. (%)	40 (9.1)	24 (16.7)	54 (12.2)	0.037	(−0.16 to 0.01)	(−0.08 to 0.02)	(−0.04 to 0.13)
Intraventricular hemorrhage grade ≥ III, no. (%)	10 (2.3)	11 (7.7)	41 (9.2)	<0.001	(−0.11 to 0.002)	(−0.11 to −0.03)	(−0.08 to 0.05)
Periventricular leukomalacia, no. (%)	10 (2.3)	6 (4.2)	7 (1.6)	0.196	(−0.06 to 0.02)	(−0.02 to 0.03)	(−0.02 to 0.07)
Retinopathy of prematurity stage ≥III, no. (%)	14 (3.6)	28 (20.4)	119 (28.0)	<0.001	(−0.25 to −0.08)	(−0.30 to −0.19)	(−0.17 to 0.02)
**Maternal**							
Maternal education more than high school, no. (%)	252 (60.9)	79 (57.3)	253 (59.8)	0.753	(−0.08 to 0.15)	(−0.09 to 0.07)	(−0.14 to 0.09)
Single parent, no. (%)	29 (6.6)	7 (4.9)	29 (6.6)	0.735	(−0.03 to 0.07)	(−0.04 to 0.04)	(−0.07 to 0.03)
Blishen score, median (IQR)	41 (33, 56)	46 (33, 57)	44 (33, 57)	0.227	(−6 to 1)	(−4 to 1)	(−3 to 4)
Maternal race Caucasian, no. (%)	274 (70.8)	93 (79.5)	280 (77.6)	0.047	(−0.19 to 0.02)	(−0.14 to 0.01)	(−0.08 to 0.12)
Maternal antihypertensive, no. (%)	101 (23.2)	14 (10.3)	75 (17.3)	0.002	(0.05 to 0.21)	(−0.01 to 0.12)	(−0.15 to 0.01)
Smoking during pregnancy, no. (%)	121 (28.3)	39 (27.7)	102 (23.8)	0.302	(−0.10 to 0.11)	(−0.03 to 0.12)	(−0.06 to 0.14)
Antenatal corticosteroids, no. (%)	378 (86.7)	116 (82.9)	366 (83.8)	0.367	(−0.05 to 0.12)	(−0.03 to 0.09)	(−0.10 to 0.08)
Maternal antibiotics, no. (%)	247 (57.2)	84 (60.4)	262 (60.5)	0.572	(−0.15 to 0.08)	(−0.11 to 0.05)	(−0.11 to 0.11)
Chorioamnionitis, no. (%)	94 (22.3)	37 (27.4)	93 (21.5)	0.354	(−0.15 to 0.05)	(−0.06 to 0.08)	(−0.04 to 0.16)
Caesarean section, no. (%)	303 (68.7)	94 (65.3)	246 (55.5)	<0.001	(−0.07 to 0.14)	(0.05 to 0.21)	(−0.01 to 0.21)

† Kruskal-Wallis test for continuous variables; Pearson χ^2^ test for categorical variables.

*Bonferroni correction applied to adjust for the three pairwise comparisons, thus confidence intervals are reported at the (1–0.05/3) % level.

BPD: bronchopulmonary dysplasia; CMV: conventional mechanical ventilation; HFO: high frequency oscillatory ventilation; NCPAP: nasal continuous airways pressure.

Maternal and neonatal characteristics are presented in **Table 2**. There were significant differences between preterm infants with no BPD, BPD, and BPD with chronic oxygen dependency for GA, BW, duration of ventilation, duration of supplemental oxygen, postnatal steroid use, diuretic use, and number of blood transfusions. Premature infants with BPD were discharged from NICU at a median of 39 weeks CA, whereas those without BPD were discharged at a median of 37 weeks (p<0.001).

**Figure 2:**
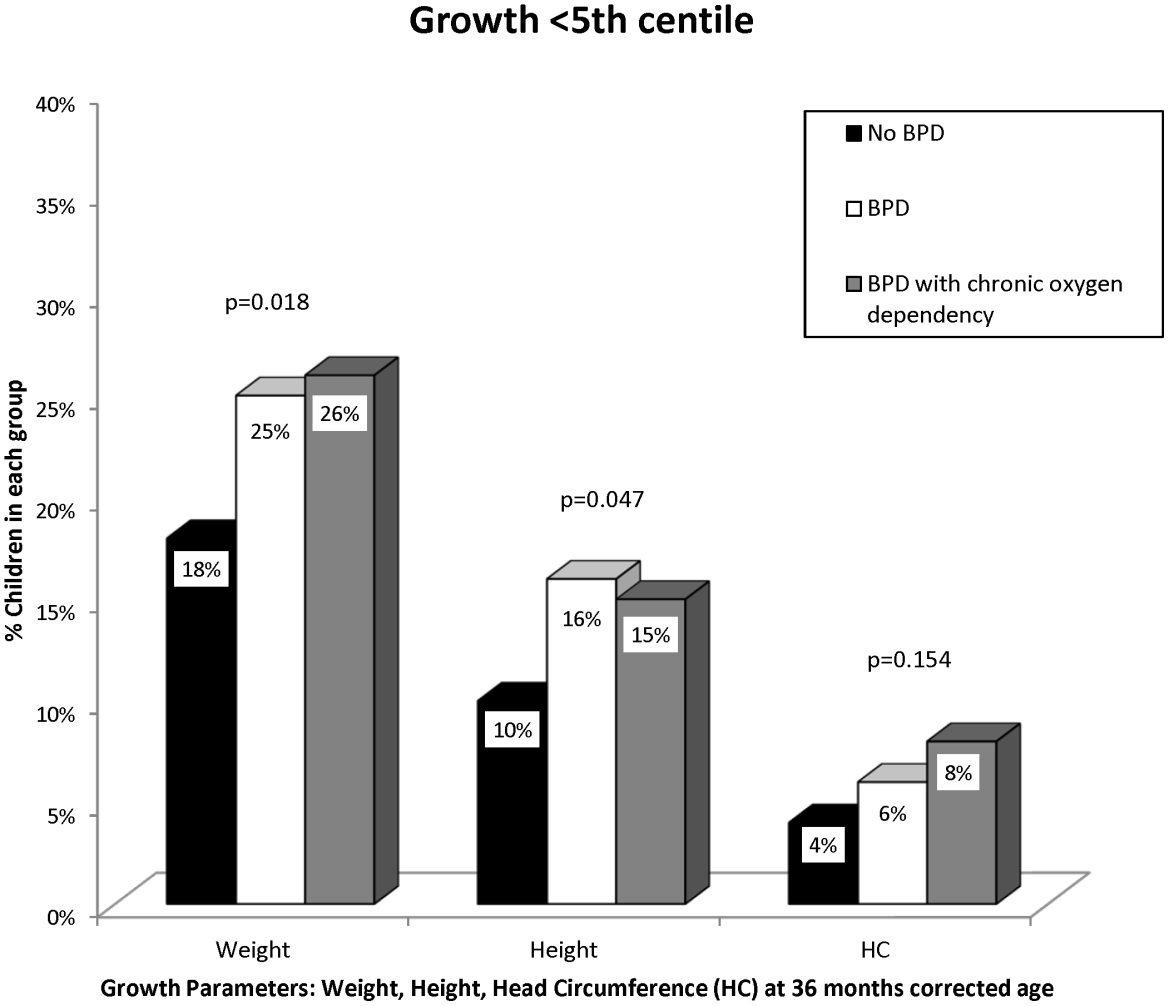
Growth parameters (weight, height and head circumference) in the No BPD, BPD and BPD with chronic oxygen dependency groups at 36 months corrected age.

**Figure 2** shows that children with BPD and BPD with chronic oxygen dependency were more likely to have weights less than the 5^th^ centile and height less than 5^th^ centile compared to children with no BPD at 36 months CA (p = 0.018 and p = 0.047, respectively). However, the proportion with head circumferences below the 5^th^ centile was not significantly different. Mean weight, height, and head circumference z-scores were not significantly different between groups (**Table 3**).

**Table 3:** Growth at 36 months corrected age.

	No BPD	BPD	BPD with chronic oxygen dependency	p-value*
Growth Parameter	(n = 409/442)	(n = 139/144)	(n = 407/444)	
Weight z-score, mean (SD)	−0.64 (1.1)	−0.83 (1.6)	−0.81 (1.3)	0.082
Height z-score, mean (SD)	−0.30 (1.0)	−0.47 (1.1)	−0.46 (1.2)	0.078
Head circumference z-score, mean (SD)	−0.08 (1.0)	−0.07 (1.2)	−0.11 (1.1)	0.892

*ANOVA F-test. Welch’s ANOVA F-test reported if variances were non-homogeneous according to Levene’s test.

BPD: bronchopulmonary dysplasia.

**Figure 3:**
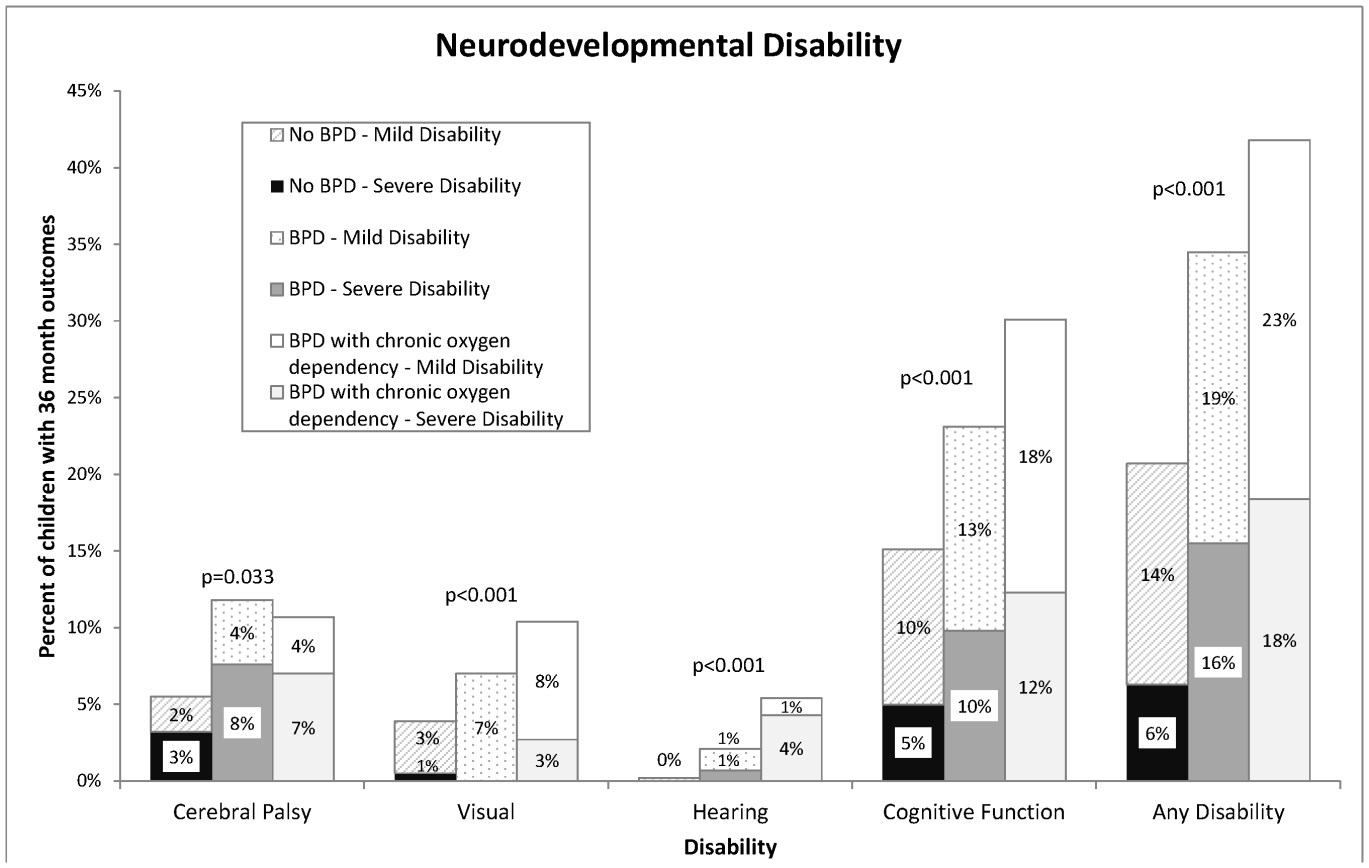
Disability Status at 36 Months Corrected Age.

Neurodevelopmental outcomes in children with no BPD, BPD, and BPD with chronic oxygen dependency are summarized in **Table 4** **and** **Figure 3**. BPD status was found to be significantly associated with disability (p<0.001). Six percent of those with no BPD had a severe disability, compared to 16% of children with BPD, and 18% of children with BPD with chronic oxygen dependency.

**Table 4:** Disability Status at 36 Months Corrected Age (No., %).

		No BPD	BPD	BPD with chronic oxygen dependency	
Disability Type	Disability Severity	n = 442	n = 144	n = 444	p-value†
Cerebral Palsy	None	418 (94.6)	127 (88.2)	396 (89.2)	0.033
	Mild	10 (2.3)	6 (4.2)	17 (3.8)	
	Moderate-Severe	14 (3.2)	11 (7.6)	31 (7.0)	
Blindness	None	422 (96.1)	133 (93.0)	395 (89.6)	<0.001
	Visual Disability	15 (3.4)	10 (7.0)	34 (7.7)	
	Legally Blind	2 (0.5)	0 (0.0)	12 (2.7)	
Deafness	None	438 (99.8)	140 (97.9)	417 (94.6)	<0.001
	Hearing Disability	1 (0.2)	2 (1.4)	5 (1.1)	
	Deaf	0 (0.0)	1 (0.7)	19 (4.3)	
Cognitive Function	Normal	372 (84.9)	110 (76.9)	307 (69.9)	<0.001
	Borderline	44 (10.1)	19 (13.3)	78 (17.8)	
	Delay	22 (5.0)	14 (9.8)	54 (12.3)	
Any Disability	None	343 (79.4)	93 (65.5)	254 (58.3)	<0.001
	Mild	62 (14.4)	27 (19.0)	102 (23.4)	
	Severe	27 (6.3)	22 (15.5)	80 (18.4)	

† Pearson χ^2^ test or Fisher’s Exact test.

BPD: bronchopulmonary dysplasia;

In the final model (**Table 5**), after adjusting for GA, maternal race, number of blood transfusions, postnatal steroids, NEC, and IVH grade 3 or 4, the proportional odds ratio (OR) for increasing disability for children with BPD with chronic oxygen dependency compared to no BPD was 1.8 (95% confidence intervals (CI): 1.1 to 2.9). The adjusted OR for increasing disability comparing children with BPD to no BPD was 1.9 (95% CI: 1.1 to 3.5). However, the adjusted OR for increasing disability comparing children with BPD with chronic oxygen dependency versus BPD was not significant at 0.9 (95% CI: 0.6 to 1.5). Replacing GA with BW in the final adjusted model gave us similar results (data not shown).

**Table 5:** Proportional odds model of factors associated with neurodevelopmental disability.

Predictors	p-value	Odds ratio (OR) for increasing disability*
BPD with chronic oxygen dependency vs. No BPD§	0.013	1.8 (1.1 to 2.9)
BPD vs. No BPD§	0.022	1.9 (1.1 to 3.5)
BPD with chronic oxygen dependency vs. BPD§	0.755	0.9 (0.6 to 1.5)
Maternal Race (Non-Caucasian vs. Caucasian)	0.005	1.8 (1.2 to 2.6)
Blood transfusions (per unit increase in no. of times)	0.001	1.1 (1.1 to 1.2)
Postnatal steroids	0.029	1.6 (1.1 to 2.5)
NEC	0.017	1.9 (1.1 to 3.1)
IVH grade III or IV	<0.001	9.6 (5.0 to 18.8)
Gestational age (per 1 week increase)	0.535	1.0 (0.9 to 1.1)

*Estimated odds ratios from proportional odds model; odds of mild or severe disability versus no disability, and odds of severe disability versus milder or no disability; Score test for the proportional odds assumption: p = 0.149; Likelihood ratio test for overall model significance: p<0.001.

§ The model was first run with No BPD as the reference category. In order to obtain estimates for BPD with chronic oxygen dependency versus BPD, the model was run a second time, using BPD as the reference category.

BPD: bronchopulmonary dysplasia; NEC: necrotizing enterocolitis; IVH: intraventricular hemorrhage; SGA: small for gestational age; PDA: patent ductus arteriosus; RDS: respiratory distress syndrome; ROP: retinopathy of prematurity.

Overall abnormal communication was not significantly different between the BPD groups (59% in BPD with chronic oxygen dependency group, 59% in BPD group, and 51% in the no BPD group) (**Table 6**). The proportion of children with abnormal receptive and expressive language was not statistically significantly different between the 3 groups.

**Table 6:** Language outcomes at 36 months corrected age.

	No BPD	BPD	BPD withchronic oxygendependency		Confidence Intervals forPairwise Differences§		
Language	(n = 385/442)	(n = 135/144)	(n = 398/444)	p-value*	No BPDvs. BPD	No BPD vs. BPDwith chronicoxygen dependency	BPD vs. BPDwith chronicoxygen dependency
Abnormal Overall Communication, no. (%)	195 (51.1)	80 (59.3)	234 (59.4)	0.053	(−0.20 to 0.04)	(−0.17 to 0.01)	(−0.12 to 0.12)
Abnormal Receptive Language, no. (%)	115 (32.9)	48 (39.3)	138 (39.3)	0.163	(−0.19 to 0.06)	(−0.15 to 0.02)	(−0.12 to 0.12)
Abnormal Expressive Language, no. (%)	144 (41.6)	55 (45.1)	158 (44.8)	0.654	(−0.16 to 0.09)	(−0.12 to 0.06)	(−0.12 to 0.13)

*Pearson χ^2^ test or Fisher’s Exact test.

§ Bonferroni correction applied to adjust for the three pairwise comparisons, thus confidence intervals are reported at the (1–0.05/3) % level.

BPD: bronchopulmonary dysplasia;

## Discussion

This study found a large number of preterm infants with BPD who were discharged home on oxygen to facilitate early transition from hospital to home from the single largest level III NICU located at high altitude in Southern Alberta. Our percentage (43%) of BPD with chronic oxygen dependency infants is lower than those reported by other investigators (60–79%) [18], but higher than other reports (36%) [19]. This could be reflective of our study population, the fact that Calgary is 1,100 m (3,600 ft) above sea level and/or our clinical practice [18], [20].

In our study, growth delay at 36 months CA was significantly more frequent in children with BPD and BPD with chronic oxygen dependency following discharge from NICU than those who had no BPD. Other investigators have also noted that impaired weight and height growth parameters occur in infants with BPD and BPD with chronic oxygen dependency [21],[22].

We found no difference in poor language outcomes between children with BPD and BPD with chronic oxygen dependency compared to children without BPD. These language findings are consistent with previous studies [23], [24].

In this study, neurodevelopmental disability, particularly cognitive impairment, at 36 months CA was significantly more frequent in children with BPD and BPD with chronic oxygen dependency following discharge from NICU than those who had no BPD. Even after adjusting for a variety of perinatal and neonatal factors, the significantly increased disability rate in children with BPD and BPD with chronic oxygen dependency versus those with no BPD still remained. To our surprise, there was no difference in outcomes, going against the pre-conceived notion that supplemental oxygen is a surrogate marker for a more severe form of BPD and is potentially contributing to increased neurodevelopmental disability even after discharge home, compared to non-oxygen dependent BPD.

While caffeine [25] and non-invasive ventilation approaches [26] are being frequently utilized early in the NICU in an attempt to decrease BPD [27], the use of supplemental oxygen in these premature infants, while significantly contributing to the pathogenesis of BPD, is a fairly constant clinical practice as it is critical for their survival [28]. While attempts have been made to limit the dose and duration of exposure of supplemental oxygen to the immature lungs [29]–[32], given the decreased capacity of the preterm infants to combat oxidative stress, it is not surprising that supplemental oxygen that is required for prolonged periods in this population results in significant medical consequences. The duration of exposure to supplemental oxygen has been associated with delayed head growth [33], and need for supplemental oxygen at 36 weeks PMA has been associated with significant neurodevelopmental delays at 12–24 months CA [34]–[37]. In contrast, a recent study has noted that BPD accompanied by invasive mechanical ventilation at 36 weeks PMA strongly predicted the more common bilateral CP phenotypes (assessed at 2 years), but BPD without invasive mechanical ventilation (i.e. only requiring supplemental oxygen at 36 weeks PMA) was not associated with any form of CP [38]. Potential explanations for neurodevelopmental disability include: excessive production of reactive oxygen species [39], fluctuations in blood oxygen levels [40]; occurrence of pneumothorax; lung dysfunction and brain injury due to infection and associated therapies [41]. Long-term follow-up studies of infants with BPD, up to 8 years of age, have reported an association with the severity classification of BPD [42] and the duration of oxygen therapy in the NICU [8]. Our finding of an increased incidence of both mild and severe CP in children who had BPD and BPD with chronic oxygen dependency is similar to Anderson and Doyle’s findings [43]. Our study supports the speculation that hypoxic brain damage and IVH ≥ grade III are more robust underlying mechanisms associated with adverse neurodevelopmental outcomes in infants with BPD and chronic oxygen dependency [7], [44].

Our data reaffirms BPD as an independent factor leading to increased neurodevelopmental disability in infants with BPD and BPD with chronic oxygen dependency, versus those with no BPD. The increased risk of neurodevelopmental disability in children with BPD/BPD with chronic oxygen dependency could be related to severe lung injury caused by prolonged hyperoxia and invasive ventilation-induced injury which possibly further exacerbates the risk of adverse neurodevelopmental outcomes secondary to severe brain injuries [10], [44]. A multi-faceted and interdisciplinary approach [45] to prevent BPD early in the course in the NICU by favoring increased antenatal steroid use [25], use of caffeine [46], non-invasive ventilation techniques [47], and enhanced nutritional support to improve growth [48], [49], offers a practical approach to also improve neurodevelopmental outcomes in such infants.

As an adjunct to clinical management of infants with BPD, home oxygen therapy at the time of discharge has been recommended in an attempt to prevent hypoxic pulmonary vasoconstriction and to allow for adequate growth [50], [51]. In our study cohort, despite almost similar median hospital stay, infants with BPD with chronic oxygen dependency versus BPD required more blood transfusions and postnatal steroids. They also had a higher incidence of PDA, IVH (grade >3) and ROP (stage ≥ III). Yet, contrary to our hypothesis, these infants with BPD with chronic oxygen dependency did not have a higher incidence of neurodevelopmental disability at 3 years CA. While earlier studies had predicted worse neurodevelopmental outcomes of infants with BPD sent home on supplemental oxygen [52], recent reports are more in line with our results [53], [54]. In one study, there was a difference in developmental scores at the 1 and 2 years follow up, but at 4 years CA, there were no differences in the BPD-room air and BPD-home oxygen groups, suggesting a catch-up between 2 and 4 years CA [53]. Taken together, these data suggest that the need for supplemental home oxygen therapy in infants with BPD does not predict increased neurodevelopmental disability over and above that of infants diagnosed with BPD at 36 weeks PMA. Importantly, even at 36 weeks PMA, it is the need for mechanical ventilation in addition to supplemental oxygen therapy that appears to be significantly associated with worse neurodevelopmental outcomes and CP. This has important implications in the prognostication of patients with BPD being sent home on oxygen therapy and has a bearing in alleviating parental anxiety in such circumstances [55].

This study has some limitations. The definitions of BPD proposed by NICHD Network [8] based on the concentrations of fraction of inspired oxygen (FiO_2_) at various postnatal ages could not be applied retrospectively. Presently, the majority of Canadian centers affiliated with the Canadian Neonatal Net Work (CNN) are using this definition. It was felt that having a similar definition at the national level would be useful to compare the outcome data and for quality improvement purposes. Management practices, especially neonatal ventilation in the NICU, changed between 1995 and 2007; however, we still had a high number of infants with BPD being discharged home on oxygen. During the study period, there were changes in institutional practices especially in increased use of antenatal corticosteroids, the type of ventilation used specifically from intermittent mandatory ventilation to patient triggered/high frequency ventilation or nasal continuous positive air way pressure (NCPAP) use, early use of parentral nutrition, caffeine prior to extubation, early PDA ligation, restricted use of blood transfusions and postnatal steroids. Since 2001, however, there has been no change regarding our policies for discharging infants home on oxygen. Postnatal steroids prescriptions in the NICU require consultation of two neonatologists and verbal consent from parents for the infants with BPD and chronic oxygen dependency with a proper documentation of risk associated with postnatal steroids use in the patients’ charts. No changes were made in the definitions of covariate morbidities and outcome measures over the period of study. We do not have information about marital status of mother and number of siblings. Blishen index reflects only the occupation of father which may not reflect actual socioeconomic status of the family. The assessors of the neurodevelopmental outcomes were not blinded to the status of BPD; hence, there is a potential risk of an assessment bias. During the study period, the Gross Motor Function Classification System was not used in our follow-up clinic to classify severity of cerebral palsy. Different cognitive tests were used over the study period and therefore, cognitive outcomes categories were used. Results of our study will be useful to the units those are located at higher altitudes [56]; therefore, generalizability is lacking especially to units located at sea level. The impact of our loss to follow-up is unknown, however, we believe it would not change the overall results as the population lost to follow-up was not significantly different in BW or GA, and a 15% loss of follow up is not an unusually high rate for such long term studies. The strength of our study is the follow-up period of 36 months. We know from past studies that 18 month outcomes are not as definitive and predictive of 3 year and 5 year outcomes. An additional strength of our study is that, to our knowledge, it is the largest single-centre, longitudinal cohort study followed prospectively that determined the impact of chronic oxygen dependency in premature infants with BPD on long-term neurodevelopmental outcomes.

## Conclusions

Chronic oxygen dependency in infants with BPD is not associated with significantly increased rates of neurodevelopmental disability at 36 months CA, compared to infants with BPD not requiring supplemental oxygen at the time of discharge home from the NICU. However, compared to children with no BPD, children with BPD and BPD with chronic oxygen dependency have a higher incidence of growth failure and cognitive delay.
